# Strategies for rapid response to emerging foodborne microbial hazards.

**DOI:** 10.3201/eid0304.970420

**Published:** 1997

**Authors:** J Majkowski

**Affiliations:** U.S. Department of Agriculture, Washington, D.C. 20250, USA. jesse.majkowski@usda.gov

## Abstract

The foodborne outbreak paradigm has shifted. In the past, an outbreak affected a small local population, had a high attack rate, and involved locally prepared food products with limited distribution. Now outbreaks involve larger populations and may be multistate and even international; in many the pathogenic organism has a low infective dose and sometimes is never isolated from the food product. Delay in identifying the causative agent can allow the outbreak to spread, increasing the number of cases. Emergency intervention should be aimed at controlling the outbreak, stopping exposure, and perhaps more importantly, preventing future outbreaks. Using epidemiologic data and investigative techniques may be the answer. Even with clear statistical associations to a contaminated food, one must ensure that the implicated organism could logically and biologically have been responsible for the outbreak.


### Strategies for Rapid Response to Emerging Foodborne Microbial Hazards[1]

**Jesse Majkowski**
U.S. Department of Agriculture, Washington, D.C., USA

The foodborne outbreak paradigm has shifted. In the past, an outbreak affected a small local population, had a high attack rate, and involved locally prepared food products with limited distribution. Now outbreaks involve larger populations and may be multistate and even international; in many the pathogenic organism has a low infective dose and sometimes is never isolated from the food product. Delay in identifying the causative agent can allow the outbreak to spread, increasing the number of cases. Emergency intervention should be aimed at controlling the outbreak, stopping exposure, and perhaps more importantly, preventing future outbreaks. Using epidemiologic data and investigative techniques may be the answer. Even with clear statistical associations to a contaminated food, one must ensure that the implicated organism could logically and biologically have been responsible for the outbreak.

### Mobilizing Resources

In the traditional paradigm of a foodborne disease outbreak, the cases were from a small local group, and the attack rate was high. Local health officials generally detected and investigated the outbreak. In the emerging paradigm, a diffuse outbreak may be spread over a very wide area, perhaps several counties or states, even with a low infective dose and a low attack rate. The outbreak may be registered only as an increase in sporadic cases and detected only because of specific laboratory-based subtyping surveillance. Whether *Salmonella* serotyping or another molecular method identifies a cluster of related cases, the investigations are more complex, and often no obvious food-handling error is found. Industry contamination may be involved, and implications may be industrywide or nationwide.

Detecting a widespread outbreak requires increased reliance on laboratory subtyping by state public health laboratories at a time when some states are considering eliminating or privatizing their laboratories. Surveillance data must be rapidly compared over increasingly broad regions, not just at the county level but at the state, regional, and national levels. Increased awareness is needed throughout the system that a local outbreak may herald a broader problem. Moreover, investigations must be conducted quickly to prevent future cases. Because of the low levels of microbiologic contamination, the right specimens and samples of food must be used. Available epidemiologic data should guide this selection so that the most likely vehicles are sampled. Traceback must extend beyond the immediate preparation of the implicated food to the whole chain of preparation, i.e., sources of ingredients, processing, storage, and transportation; cooperation at all levels of industry is required. The goals are to control the ongoing outbreak, remove the contaminated product from the market, and learn how to prevent similar outbreaks. Intervention in outbreaks must change. Emergency intervention must be based on solid epidemiologic data (appropriate study design and sample size and clear statistical association with logical and biologic plausibility) and cannot always wait for laboratory confirmation of a contaminated product. Illnesses will not wait for laboratory examination to yield the pathogen; the pathogen may not be detectable with current technologies, the food may not be available, and the delay can be critical.

Address for correspondence: Jesse Majkowski, U.S. Department of Agriculture, Room 3715 Franklin Court, 14th Street & Independence Avenue, S.W. Washington, DC 20250 USA; fax: 202-501-7515; e-mail: jesse.majkowski@usda.gov.

[1]The author has summarized the transcripts of panel discussions by Dennis Lang, Craig Hedberg, Eric Johnson, Suzanne Binder, and Philip Tarr.

### The Human Side of Foodborne Disease

Public health officials in Washington state screened children with bloody diarrhea and hemolytic uremic syndrome (HUS) as the result of a large outbreak of *Escherichia coli* O157:H7 infection in 1993. One isolate of the organism caused perhaps 75% of the cases, not 100%. As a result, the source of the infection was identified, and regulatory action was taken to halt future cases. Hospitals reported fewer new cases after this action. However, 500 Washington state residents, two-thirds of them children under 15 years of age, became infected before the incriminating food could be removed from the market. The HUS attack rate was approximately 12% for children under the age of 16 years. The organism was recovered from the food product (hamburger), and DNA fingerprinting was initiated by several techniques. The number of colony-forming units of *E. coli* O157:H7 in the hamburger was relatively low.

This organism can cause severe life-threatening infection, even with an inoculum rate too low to be detected by testing. In the United States, the incidence of HUS is approximately 1.7 cases per 100,000 children under the age of 15 years. This figure is based on data from King County, Washington, in the early 1980s and indicates that there are 1,000 cases of HUS in the United States per year or an average of 2.8 cases per day in a population of 250 million. The Centers for Disease Control and Prevention has recently reported that only half of the country's microbiologists screen for this organism.

### *Clostridium botulinum*, A Reemerging Pathogen

The outstanding property of *C. botulinum* is its ability to synthesize a neurotoxin of extraordinary potency (lethal dose is approximately one microgram). *C. botulinum* is an unusual foodborne pathogen in that it causes neuroparalytic rather than diarrheal disease. During illness, the first nerves affected are the cranial nerves in the head and eye; the paralysis can descend and affect every peripheral nerve in the body. The toxin can enter into foods, but in recent years, *C. botulinum* has also been found to colonize the intestinal tract of infants under 1 year of age and of adults that have undergone intestinal surgery or have been treated with antibiotics. The number of cases of adults with unusual clostridia that produce botulinum toxin is increasing.

Botulism occurs worldwide. The highest incidence is found in Poland and in Asia and is related to food handling (in Poland, home canned meats).

New food processes and packaging have been associated with the reemergence of botulism. A clam chowder outbreak in California involved a boxed food that was not properly stored. Because boxed foods generally do not require refrigeration, the food was kept at ambient temperature; however, it was not shelf stable and should have been refrigerated.

A large outbreak (30 cases) of botulism occurred in a restaurant in El Paso when potatoes were cooked in foil, left wrapped, and then used in potato salad. Baking had eliminated vegetative organisms, but the spores of botulinum were not killed. In this case, a low-acid food was held under anaerobic conditions.

Botulism outbreaks are probably the most reported type of foodborne illness. Changes in processing and ingredients in foods and formulations can inadvertently lead to the growth of *C. botulinum*. Failure to thoroughly heat a food product may allow the botulinum toxin to survive.

### DNA Fingerprinting

Responding to the threat of emerging foodborne diseases requires public health surveillance that is based on epidemiologic methods and close collaboration between epidemiologists and public health laboratories. Surveillance for foodborne diseases should include pathogen-specific surveillance to identify clusters of cases caused by the same organism and epidemiologic investigations to identify the source.

The Minnesota State Department of Health is developing a new approach to foodborne disease surveillance. A *Salmonella* Enteritidis (SE) outbreak was first recognized by the public health laboratory when the number of SE isolates suddenly increased. Because many of the isolates came from clinical laboratories in southeastern Minnesota, the outbreak initially seemed regional. However, within 48 hours of initiating a case-control study, a nationally distributed food product was identified as the vehicle, and the nationwide scope of the outbreak was documented. This was an example of consequential epidemiology. When the association between food consumption and SE was announced, the evidence implicating a particular brand was limited to a single case-control study of 15 matched pairs. Laboratory isolation of SE from official samples was not reported until 10 days later. This prompt action, based on epidemiologic data, prevented at least 10 days of potential exposure for thousands of consumers. Consequential epidemiology produces results that translate into disease prevention. We need to continue to develop models for how to rapidly evaluate and act on epidemiologic data and how to better coordinate activities regionally and nationally.

A critical element of the success of this type of foodborne disease surveillance is the specificity with which we can match isolates that may be epidemiologically linked. Molecular subtyping schemes such as pulsed-field gel electrophoresis can greatly improve identification of clusters of the same organisms such as particular *Salmonella* serotypes.

Another example involved analysis of a typical epidemiologic curve for a seemingly single outbreak. When isolates were analyzed, however, a cluster of small outbreaks was found. One was caused by infected food handlers at a restaurant. This outbreak would have continued, and the infected food handlers would have provided an ongoing source of infection to patrons had the cluster not been identified through subtype-specific surveillance. This incident serves as a model for how foodborne disease surveillance systems must be developed and used. Molecular subtyping has revolutionized our ability to conduct meaningful surveillance. We consider it an integral part of disease prevention and control and continue to explore its usefulness.

### Parasites

*Cyclospora cayetanensis* is a protozoan coccidian parasite. A one-celled organism, it is related to other organisms such as *Toxoplasma* and *Cryptosporidium*. It is a prototypical emerging pathogen. *C. cayetanensis* is unusual in that it is not immediately infectious when excreted. Under optimal conditions, it matures in days to weeks, so direct person-to-person spread is very unlikely. An outbreak following a meal is probably not caused by the food handler. The organism appears to be seasonal, and in most places where it has been studied, it occurs in the spring or summer and causes little or no disease during the fall or winter. Infection has been reported throughout the world, and the key studies have been conducted in Peru and Nepal. Disease caused by *C. cayetanensis* is characterized by watery stools, nausea, weight loss, low-grade fever, fatigue, or any combination of these symptoms. The disease (which is easily treatable) can be quite protracted, and without treatment, relapse can occur. The mean incubation period of 1 week complicates the epidemiology; cases may not be recognized until 2 weeks after people have been exposed.

In 1996, more than 1,450 cases of *Cyclospora* were reported in the United States (87%) and Canada (13%). Approximately half of them were in clusters; the other half were sporadic (not epidemiologically linked to other cases). More than 65% of the 1,450 cases were laboratory confirmed; 22 infected patients were hospitalized. Fifty-five clusters were reported, 47 in the United States and 8 in Canada. An average of 28 attendees per event and a very high attack rate were reported. The attack rate was 56% for attendees, not for people who ate the implicated food. At least one type of fresh berry was served at every event and, despite other types of exposures, no other food was implicated in any cluster investigation. The berry did not always achieve statistical significance, largely because of the small number of attendees at a specific event. The berry most likely linked to the cases was later determined. That type of berry was served at 50% to 91% of the 55 events; it was the only kind served at 10 or 11 of the events.

We had overwhelming epidemiologic evidence, but we never identified *Cyclospora* on any raspberries. Two factors, at least, contributed to this. The test for *C. cayetanensis* did not exist when this outbreak occurred; therefore, no implicated raspberries were tested. The question in this investigation as in others is how much epidemiologic evidence is needed to implicate a food as the vehicle of disease? A review team may be needed to look at the epidemiologic data and determine if they are adequate to warrant informing the public about a hazardous food. We are seeing new pathogens, new species. As outbreaks cross into other states, the need for coordination between health officials in the states and in the federal government becomes more urgent.

### Conclusions

Early identification of the outbreak and the organism can prevent future cases. Recent investigations have found that the presence of an organism even at low levels can cause serious consequences. Fingerprinting organisms for identification during outbreaks is extremely important. In some instances, fingerprinting has helped identify several small outbreaks that initially appeared to be one large outbreak. We can no longer afford to wait for all the evidence and laboratory results to be collected and reported; we must use epidemiologic data. Once the outbreak is identified, DNA fingerprinting is needed to identify whether other outbreaks are occurring simultaneously.

A rapid and coordinated response is needed among state officials and federal agencies. Interventions should stop outbreaks and identify products causing illness so they can be removed from the market. Then health officials need to take the next step—investigate what happened and determine the cause so that similar outbreaks can be prevented.

